# Entropy Wake Law for Streamwise Velocity Profiles in Smooth Rectangular Open Channels

**DOI:** 10.3390/e22060654

**Published:** 2020-06-13

**Authors:** Domenica Mirauda, Maria Grazia Russo

**Affiliations:** 1 School of Engineering, Basilicata University, Viale dell’Ateneo Lucano 10, 85100 Potenza, Italy; 2 Department of Mathematics, Computer Science and Economics, Basilicata University, Viale dell’Ateneo Lucano 10, 85100 Potenza, Italy; mariagrazia.russo@unibas.it

**Keywords:** Shannon’s information entropy, wake law, streamwise velocity profiles, velocity-dip-position, open channel flows, laboratory experiences, error analysis, rectangular cross-section

## Abstract

In narrow open channels, the three-dimensional nature of the flow and the transport momentum from the sidewalls to the central region cause the maximum longitudinal velocity to occur below the water surface. The entropy model is unable to accurately describe the velocities near the free surface when the dip phenomenon exists. The present paper proposes a new dip-modified entropy law for steady open channel flows, which consists of three additional terms: the first one similar to Coles’ function; the second one linearly proportional to the logarithmic distance from the free surface; and the third one depending on the cubic correction near the maximum velocity. The validity of the new model was tested on a set of laboratory measurements carried out in a straight rectangular flume with smooth boundaries and for different values of water discharge, bottom slope, and aspect ratio. A detailed error analysis showed good agreement with the data measured through the present research and a more accurate prediction of the velocity-dip-position compared with the one evaluated through the original entropy model. In addition, the modified entropy wake law matched very well with other literature data collected in rectangular cross-sections with different flow conditions.

## 1. Introduction

Over the past century, the knowledge of velocity vertical distribution in an open channel cross-section received great attention by several research teams due to its importance in the understanding of numerous hydraulic phenomena such as flood control, pollutant dispersion, and sediment transport, and its various applications in the design and planning of hydropower plants and structures or infrastructures interacting with fluid flows [1,2,3,4,5,6,7,8]. The sophisticated numerical modeling and advanced experimental techniques allowed for obtaining a good reconstruction of velocity profiles in fully-developed turbulent wide open channel flows, though they have yet not been able to well describe the dip of the maximum velocity below the free surface in narrow open channels due to the presence of secondary currents [9,10,11,12,13]. In the latter conditions, in fact, the classical log or power laws, which depict the velocity increasing monotonically with the distance from the bottom, deviate from the experimental results and fail to predict the dip phenomenon [14]. Coles [15] was among the pioneers to investigate this deviation, adding a purely empirical correction term, called wake function, to the log law. Later, some researchers tried to adapt Coles’ wake function to experimental data, suggesting different empirical values to estimate the parameters within this law [10,11,16,17,18]. In particular, Nezu and Rodi [10] found Coles’ profile parameter *II* to be between 0 and 0.20 by analyzing the longitudinal and vertical velocity components in two-dimensional, fully developed open channel flows over smooth beds. Cardoso et al. [11] claimed that the wake strength was dependent on the secondary currents, flow history, and inactive turbulence components and yielded a *II* value equal to −0.077 for hydraulically smooth conditions. By studying the influence of the suspended sediment on the shape of the velocity distributions, Coleman [16] obtained an average value of *II* equal to 0.19. For fully developed, rectangular, subcritical open channel flows on smooth beds, Kirkgoz [17] reported a constant value of this parameter of approximately 0.1, considerably smaller than the one given by Coles (*II* = 0.55). Kironoto and Graf [18] showed how the profile parameter was affected by the aspect ratio *A_r_ = b/D* (channel width/flow depth) and the boundary conditions, finding a value of 0.09 for rough plate data with *A_r_* > 5 and −0.03 for gravel bed data with *A_r_* < 5.

Other researchers proposed various improvements to the log and wake laws to better predict the velocity-dip phenomenon in open channels flows [19,20,21,22,23,24,25,26,27,28,29,30,31].

In 2000, Sarma et al. [19] introduced a binary velocity profile model, which combines the logarithmic law of the inner region with the parabolic law of the outer region for subcritical and supercritical flows in smooth and rough channels. They found that the junction point of the two curves depended on the width and aspect ratios of the cross-section and was 0.5 *D* (where *D* is the water depth) when there was no dip phenomenon and decreased from 0.5 *D* to 0 in the presence of the dip phenomenon.

Wang et al. [20] demonstrated the validity of log and wake laws also for sediment-laden flows and they applied a regression analysis to study the influence of the main factors, such as the Karman constant and the profile parameter, on the vertical velocities. By comparing various experimental literature data, they observed how the maximum velocity was affected by the aspect ratio, *A_r_*, and, in particular, it was located in the central portion and below the free surface for narrow channels, while it moved towards the sidewall region and near the free surface for wider channels.

A modified log-wake law (MLW-law) was developed by [21,22,23] by introducing a cubic function that well represented the deviation of the experimental data in pipes and boundary layers where a zero-velocity gradient exists at the maximum velocity. Later, the same authors successfully extended the application of the modified law to turbulent open channel flows of the laboratory and field, although it could not be a universal model due to the presence of empirical parameters [22,24].

Yang et al. [25], instead, derived the dip-modified log law (DML-Law) from the analysis of the Reynolds-averaged Navier–Stokes (RANS) equations. This law had the great advantage of containing the unique parameter α for dip correction, even though it was able to accurately reconstruct the velocities along the verticals from the central line to the sidewalls only in uniform open channel flows with smooth boundaries. The extension to flow regimes with rough walls was performed by Absi, who first introduced a simple dip-modified log-wake law in 2009 [26] and then a full dip-modified log-wake law (fDMLW-law) in 2011, both based on a log-wake-modified eddy viscosity distribution [27]. The fDMLW-law was further modified by [28] in 2012 in order to estimate the velocity profile throughout the depth of the channel without relying on numerical integration.

At the same time, Wang and Cheng [29] proposed a modified streamwise profile velocity similar to the log-wake law but varying periodically in the lateral direction and based on the assumption of zero turbulent shear stress at the maximum velocity location to describe the secondary flows artificially generated with alternate rough and smooth channel bed strips.

On the basis of experimental observations, Bonakdari et al. [30] developed a sigmoid model to describe the dip phenomenon in the outer region of smooth narrow as well as wide channels, which was a function of the *A_r_*. Although this model did not overestimate the measured velocities in the central cross-section like those of [20,25], it did not satisfy the asymptotic boundary conditions in which the maximum velocity occurred at 0.5 *D* for *A_r_*→0 and at 1 *D* for *A_r_*→∞. This is due to the fact that the model was obtained to fit the experimental data of ovoid-shaped sewers [31,32].

Later, Lassabatere et al. [33] derived a new law for the streamwise velocity profile in the outer region of the central section of open channels by integrating the Reynolds-averaged Navier–Stokes equations with the analytical modeling of the vertical component and by adopting a negligible viscosity. This law, tested on several experimental datasets that include rough and smooth flow regimes, satisfied both the asymptotic boundary conditions, unlike the previous models.

In recent decades, apart from the log law and the log-wake law, the power law was proposed to describe the velocity distribution in most pipe flows, boundary layers and wall jets, especially in the overlapping region of the inner law (i.e., the law of the wall) and the outer law (i.e., the velocity defect law) [34]. Already in the late fifties, various researchers [35,36,37] had shown how the power velocity profile was preferable to the logarithmic velocity distribution. In particular, it could be applied to different flow regimes and Reynolds numbers [34], it seemed better at incorporating the effects of sediment without singularities near the bed and/or discontinuities at the axes and planes of symmetry [34,38], and it better agreed with the pipe flow data [37,39].

In 2007, Cheng [40] derived the power law as a first order approximation to the log law and was able to reconstruct the velocity profile in the overlap layer between the inner and outer regions of open channel flows. He found that its exponent was a function of the Reynolds number, the relative roughness height, and the friction factor, and that it exceeded the value of 1/6 in the presence of large-scale boundary roughness, as already observed by [34].

Afzal et al. [41,42] proposed the envelope of the skin friction power law to study the velocity distribution in fully developed turbulent pipe and channel flows. Although the results of this model were not very different from the ones obtained with the log law for high Reynolds numbers, they were able to better reconstruct the velocity profiles for low Reynolds numbers.

In 2009, Castro-Orgaz [43] derived an analytical approach by integrating the von Karman momentum equation using a power velocity distribution inside the boundary layer flow in the turbulent rough regime. The analytical solutions proved to be a close fit to the experimental data of flows over uncontrolled spillway crests followed by a steep chute of constant slope.

The aforementioned deterministic approaches based on theoretical predictions or empirical evaluations of variables and parameters are often questionable and do not account for the randomness included in time-averaged streamwise velocities. To overcome these limitations, the informational theory, using Shannon’s entropy [44] and the principle of maximum entropy (POME) [45,46,47], was applied first by Chiu [48] in order to discuss the uncertainties associated with the flow field. This theory was also used to study different hydraulic phenomena, such as the distribution of the boundary shear stresses [49,50,51,52] and suspended sediment concentrations [53,54,55,56,57] in open channel flows. The entropy law has the advantages of satisfactorily predicting the velocity near the bed and of relying on the estimation of a single discriminating parameter, depending on the ratio between the mean and maximum velocity of the investigated cross-section. This parameter seems to keep constant over the entire section despite the varying water discharge in [58,59,60,61] and over the entire reach for rivers with the same morphological characteristics in [62]. As a consequence of its application, such uniformity induces the simplification of the numerical modeling and the reduction of the sampling time during the water discharge measurement in rivers by predicting the value of the mean velocity from the knowledge of the maximum velocity only [61]. However, the entropy model deviates from the near-free-surface velocities in narrow open channels when the maximum velocity occurs below the water surface due to the presence of secondary currents and wall effects, as widely observed for the classical logarithmic and power laws [63,64,65]. The present paper proposes a new dip-modified entropy law in order to reconstruct the entire velocity profile and predict the dip phenomenon for low values of the aspect ratio. This law was first validated using measurements collected in this research on a rectangular laboratory channel, in conditions of steady flow and smooth boundaries and for different water discharges, bottom slopes, and aspect ratios, and was then tested on a set of literature data. A detailed error analysis was applied on the observed and calculated velocities to demonstrate the good performance of the modified model compared with the original one.

## 2. Experimental Set-Up

**Figure 1:**
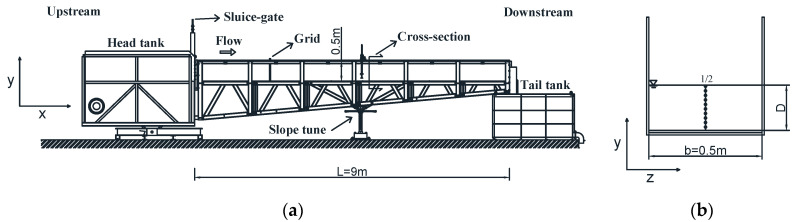
Experimental set-up: (**a**) smooth rectangular open channel; (**b**) investigated cross-section.

The experiments were performed in a 0.5 m wide, 0.5 m deep, and 9 m long rectangular flume in the Hydraulics Laboratory at the School of Engineering, Basilicata University (Italy). The channel was connected to a head tank, which regulates the flow at the entrance as much as possible, and a tail tank, which allows the water recirculation through a pipes and pumps system. The sidewalls were made of glass to facilitate the flow visual observations, while the bottom was of plexiglass in order to obtain the hydraulically smooth condition. The layout of the experimental set-up is shown in Figure 1. The investigated cross-section was chosen in the middle of the flume in order to observe a fully developed turbulent flow, avoiding edge effects. A honeycomb was also located upstream of the same section to make the velocity distributions uniform.

**Figure 2:**
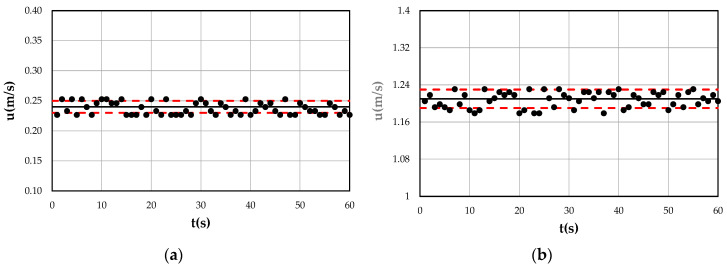
Two examples of (**a**) low and (**b**) high point velocity trends acquired with micro current meter.

The water discharge was measured with a concentric orifice plate installed in the feed pipe with a 5% smaller error. The flow depth was measured by two hydrometers (twin wire wave probe—600 mm of HR Wallingford) placed at both the beginning and the end of the measurement cross-section, and was assumed as the average value. A series of velocity profiles was obtained by a micro current meter (Nixon Instrumentation Mod. 403 *u* = 5–150 cm/s, and Mod. 404 *u* = 30–300 cm/s) in the central line with different aspect ratios, *A_r_,* (3.0–9.0), flow depths, *D*, (0.053–0.204 m), water discharges, *Q*, (0.01–0.095 m^3^/s), and bottom slopes, *i* (0–1%). Figure 2 shows two examples of low and high values of point velocity acquired with the micro current meter with varying time. As can be seen, the standard deviation of both signals is in the range 0.01–0.02 m/s, which is of the same order of accuracy as the instrument, underlining the precision of the measurement technique. This precision has been further confirmed by the constancy of the mean value and variance with varying time (Table 1). The velocity measurements were mainly taken at 5 mm above the bed and at every 0.005 m interval up to 0.01 m below the free surface. In particular, the difference between the two acquired consecutive velocities was always less than 10% of the maximum value, thus applying a more accurate criterion than the one proposed in the ISO 748/1997 [66]. In Table 2, the ranges of the flow characteristics of the laboratory tests are shown.

## 3. The Proposed Model

### 3.1. Shannon’s Entropy-Based Velocity Distribution

By defining a new coordinate system in connection with the probability and space domain and stemming from a probabilistic approach, Chiu [48] derived the time-averaged velocity, $\tilde{u}$, along the vertical of an open channel cross-section, considering it as a random variable associated to the probability density function (PDF), $f\left(\tilde{u}\right)$*,* equal to

(1) $$\frac{\xi -\xi_{0}}{\xi_{max}-\xi_{0}}=\int_{0}^{u}f\left(\tilde{u}\right)d\tilde{u},$$

where *ξ* is the dimensionless variable which depends on the reference system used for the local representation of the flow field, while *ξ*_0_ and *ξ_max_* are the values of the dimensionless variable corresponding to the minimum ($\tilde{u}=0$) and the maximum ($\tilde{u}=u_{max}$) velocity, respectively.

In Equation (1), the last-biased PDF can be obtained by the maximization of Shannon’s entropy according to Jaynes [45,46,47]:

(2) $$H=-\int_{0}^{u_{max}}f\left(\tilde{u}\right)lnf\left(\tilde{u}\right)d\tilde{u},$$

and applying the following constraints:

(3) $$\int_{0}^{u_{max}}f\left(\tilde{u}\right)d\tilde{u}=1,$$

(4) $$\int_{0}^{u_{max}}\tilde{u}f\left(\tilde{u}\right)d\tilde{u}=u_{mean}.$$

One simple way to achieve the maximization of *H* is using the method of the Lagrange multipliers. To that end, the Lagrange function *L* can be constructed as

(5) $$L=-f\left(\tilde{u}\right)lnf\left(\tilde{u}\right)+\lambda_{1}f\left(\tilde{u}\right)+\lambda_{2}\tilde{u}f\left(\tilde{u}\right).$$

Differentiating Equation (5) according to $f\left(\tilde{u}\right)$ and equating the derivative to 0, one gets

(6) $$\frac{\partial L}{\partial f}=-lnf\left(\tilde{u}\right)-1+\lambda_{1}+\lambda_{2}\tilde{u},$$

from which the probability density function, $f\left(\tilde{u}\right)$, including the Lagrange multipliers, is obtained:

(7) $$f\left(\tilde{u}\right)=exp\left(\lambda_{1}-1\right)exp\left(\lambda_{2}\tilde{u}\right).$$

The two Lagrange multipliers, *λ*_1_ and *λ*_2_, can be derived by substituting Equation (7) in Equations (3) and (4):

(8) $$e^{\lambda_{1}-1}=\lambda_{2}{\left(e^{\lambda_{2}u_{max}}-1\right)}^{-1},$$

(9) $$u_{mean}=u_{max}e^{\lambda_{2}u_{max}}{\left(e^{\lambda_{2}u_{max}}-1\right)}^{-1}-\frac{1}{\lambda_{2}}.$$

With *f(u)* represented by Equation (7), Equation (1) can be integrated to yield the form of the entropy velocity profile [67]:

(10) $$u=\frac{1}{\lambda_{2}}ln\left(1+\frac{\lambda_{2}}{e^{\lambda_{1}-1}}\frac{\xi -\xi_{0}}{\xi_{max}-\xi_{0}}\right).$$

In order to simplify the velocity distribution equation, Chiu [68] introduced a new dimensionless parameter defined as

(11) $$M=\lambda_{2}u_{max}.$$

The entropic parameter, *M*, is an effective measure of the overall characteristics of a cross-section, as represented by the bed material, slope, shape, and alignment, and can be used to classify various channel sections and their equilibrium state [69]. It is linked to the ratio between the mean and maximum velocity of the cross-section through Equation (9):

(12) $$\frac{u_{mean}}{u_{max}}=e^{M}{\left(e^{M}-1\right)}^{-1}-\frac{1}{M}.$$

Substituting the Lagrange multiplier *λ*_2_ and calculating the term $e^{\lambda_{1}-1}$ through Equation (8), Equation (10) can be expressed in the following form:

(13) $$\frac{u_{mean}}{u_{max}}=\frac{1}{M}ln\left[1+\left(e^{M}-1\right)\frac{\xi -\xi_{0}}{\xi_{max}-\xi_{0}}\right].$$

Equation (13) describes the velocity profile in the central axis of open channels where the maximum velocity, *u_max_*, can occur on or below the water surface. In wide channels, *(ξ* − *ξ*_0_*)/(ξ_max_* − *ξ*_0_*)* can be replaced by *y/D* [68]. For narrow channels, when the aspect ratio is low (e.g., lower than 5 for [70]), the maximum velocity is generally below the free surface and the term *(ξ* − *ξ*_0_*)/(ξ_max_* − *ξ*_0_*)* can be defined as

(14) $$\xi =\frac{y}{D-h}exp\left(1-\frac{y}{D-h}\right),$$

where *h* is depth below the water surface in which the maximum of the velocity is observed, and *y* the vertical distance from the channel bed.

Experimental studies by [71] proved that, for channels at different shapes of the cross-section, the maximum velocity generally occurs below the free surface, specifically around 25% of the maximum flow depth. In such conditions, the value of *h* can be assumed equal to 3/4 of the maximum depth and the variable ξ becomes

(15) $$\xi =\frac{4}{3}\frac{y}{D}exp\left(1-\frac{4}{3}\frac{y}{D}\right).$$

### 3.2. A New Entropy-Wake Law

**Figure 3:**
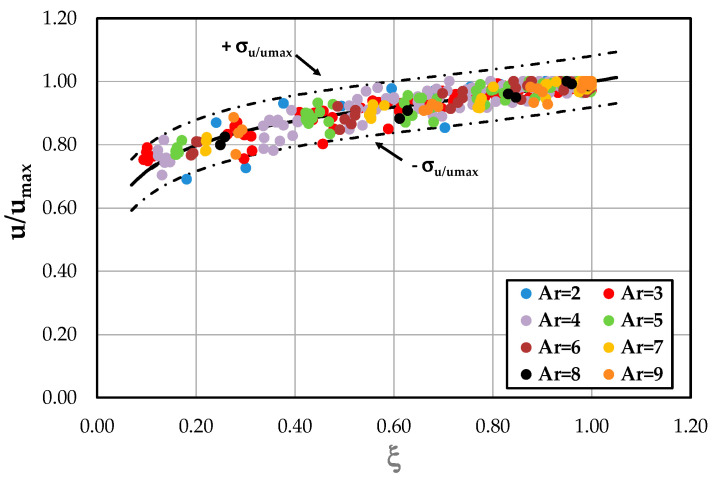
Validation of the proposed law by the experimental data.

Various studies of the literature show how the entropic profile does not predict the near-free-surface velocities in open channel flows with secondary currents and where the sidewall effects are not negligible [63,64,65], as extensively discussed for the classical logarithmic and power laws [19,20,21,22,23,24,25,26,27,28,29,30,31,32,33,34,35,36,37,38,39,40,41,42,43]. In order to research a modified entropy model able to fit the experimental data in the outer region *y/D* > 2, a nonlinear least square approximation scheme (@MATLAB function fit with the Levenberg–Marquardt approach) was applied. This allowed obtaining a new entropy velocity distribution equal to

(16) $$\frac{u}{u_{max}}=\frac{1}{M}ln\left[1+\left(e^{M}-1\right)\frac{\xi -\xi_{0}}{\xi_{max}-\xi_{0}}\right]+\alpha sin^{2}\left(\frac{\pi }{2}\xi \right)+\alpha ln\left(\xi \right)-\alpha \xi^{3},$$

where the value of *α*, empirically obtained, was equal to −0.04 after fitting Equation (16) with the experimental data of Table 2 (Figure 3). Equation (16) is constituted by a wake function similar to Coles’, a term linearly proportional to the logarithmic distance from the free surface in order to account for the three-dimensional nature of the flow in open channels with secondary currents [26,27], and by the cubic function, in order to adapt the profile to the maximum velocity [21,22,23,24].

As can be seen, the proposed law well agrees with the experimental results since 98.4% of the data fall within the 95% confidence interval.

**Figure 4:**
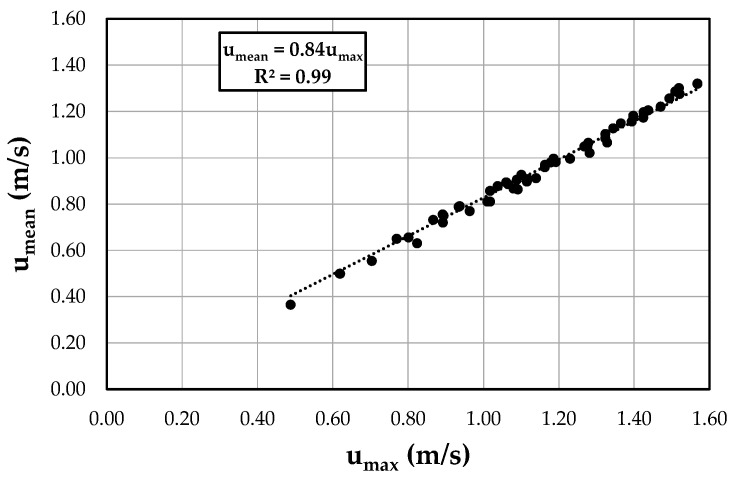
Relation between the experimental mean and maximum velocities with varying water discharge.

In Equation (16), a constant entropic parameter, *M* = 6.10, was used for the whole data set obtained from the best-fit line of the mean and maximum velocities in the investigated cross-section with varying water discharge (Figure 4). The high correlation confirms the channel section tendency to establish and maintain an equilibrium state under a wide range of flow conditions to which a single value of velocity entropy corresponds [72]. The mean and maximum velocities were obtained in a more accurate way compared with the ISO 748/1997 [66], as described in the experimental set-up.

## 4. Discussion of Results

**Figure 5:**
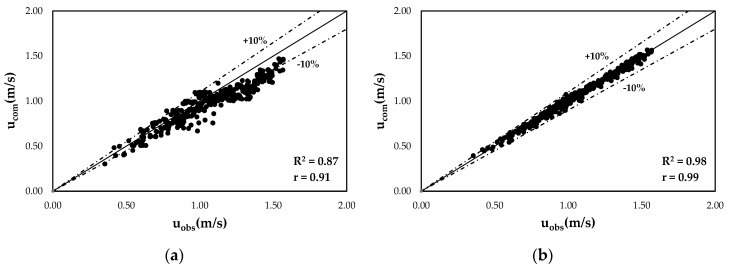
Comparison between the observed, *u_obs_*, and the computed, *u_com_*, velocities through: (**a**) the classical entropy model; (**b**) and the entropy wake law.

The reliability of the proposed model is demonstrated by the comparison of the velocities computed through the entropy wake law and original entropy law with the ones observed in the open channel cross-sections for each value of water discharge, aspect ratio, and bottom slope (Table 2), reported in Figure 5.

As one can see from Figure 5, high values of the determination coefficient and of Pearson’s correlation coefficient underline a perfectly positive linear relationship between the calculated and the observed data and less error variance in the latter specified by the proposed model. The velocities are reproduced fairly well by the proposed law for the investigated range of water discharges, bottom slopes, and aspect ratios, showing an error within ±10% for the 99.5% of the observed velocities, while in the case of the classical model, only 66.3% of the observed velocities fall within the 10% error band.

In addition, a detailed error analysis using different statistical indices, such as the root mean square error (RMSE), the RMSE observations standard deviation ratio (RSR), the mean absolute error (MAE), the percentage of bias (PBIAS), and the Nash–Sutcliffe efficiency (NSE), was considered to quantify the good performance of the new law [73]:

(17) $$\mathrm{RMSE}=\sqrt{\frac{1}{n}\overset{n}{\underset{i=1}{\sum }}{\left[\frac{u_{\left(com\right)i}-u_{\left(obs\right)i}}{u_{\left(obs\right)i}}\right]}^{2}},$$

(18) $$\mathrm{RSR}=\frac{\sqrt{\sum_{i=1}^{n}{\left[u_{\left(com\right)i}-u_{\left(obs\right)i}\right]}^{2}}}{\sqrt{\sum_{i=1}^{n}{\left[u_{\left(obs\right)i}-u_{\left(mean\right)i}\right]}^{2}}},$$

(19) $$\mathrm{MAE}=\frac{1}{n}\overset{n}{\underset{i=1}{\sum }}\left|u_{\left(com\right)i}-u_{\left(obs\right)i}\right|,$$

(20) $$\mathrm{PBIAS}=\left[\frac{\sum_{i=1}^{n}\left[u_{\left(obs\right)i}-u_{\left(com\right)i}\right]\cdot 100}{\sum_{i=1}^{n}u_{\left(obs\right)i}}\right],$$

(21) $$\mathrm{NSE}=1-\left[\frac{\sum_{i=1}^{n}{\left[u_{\left(obs\right)i}-u_{\left(com\right)i}\right]}^{2}}{\sum_{i=1}^{n}{\left[u_{\left(obs\right)i}-u_{\left(mean\right)i}\right]}^{2}}\right],$$

where *u_com_* and *u_obs_* are the computed and observed time-averaged velocities along the central vertical of the investigated cross-section, respectively.

The RMSE and the MAE, depending on the presence of outliers and the shape of the error distribution, have the advantage of showing the difference between the predicted and the observed values in the same units. Values of the RMSE and MAE close to zero highlight higher model accuracy. The RMSE can be substituted by its standardized version, RSR, which includes the standard deviation of the measured data as the scaling/normalization factor, and still incorporates the benefits of the RMSE itself [74].

The PBIAS measures the average tendency of the calculated data to be higher or lower than the observed ones [75]. The null value of PBIAS indicates an accurate model simulation, while its positive and negative values indicate a model over- and underestimation, respectively. The NSE is a dimensionless technique, which determines the relative magnitude of the residual variance when compared with the observed data variance [76]. The values of the NSE range from −∞ to 1.0, with NSE = 1 as the optimal value. When the NSE is between 0 and 1, the levels of performance are acceptable, while with the NSE lower or equal to 0, the performance is unacceptable. These last three indices allow assessing the accuracy of the proposed model according to the four categories defined in Table 3 [77].

Table 4 shows the comparison between the suggested statistics of the velocities obtained from the entropy wake law and the ones evaluated through the classical entropy law. As seen in the table, the model performance goes from satisfactory to very good. This underlies how the new formula provides a very accurate and reliable estimation of the velocities along the entire vertical from the bottom to the free surface.

A further validation of the proposed model was carried out using the literature data of [16,78], and [79]. The authors collected point streamwise velocities both with clear water and with sediment-laden flows in rectangular flumes with varying slope, water discharge, flow depth, and aspect ratio in order to analyze the influence of the suspended sediment on the shape of the velocity profiles. In this work, only the runs conducted in the condition of clear water are used (Table 5). Coleman [16] measured the velocity distributions by a Pitot-static tube, while Lyn [78] as well as Muste and Patel [79] used laser Doppler velocimetry.

Table 6 underlines the very good performance of the proposed new law also for the literature data, while the classical entropy model is often unsatisfactory.

**Figure 6:**
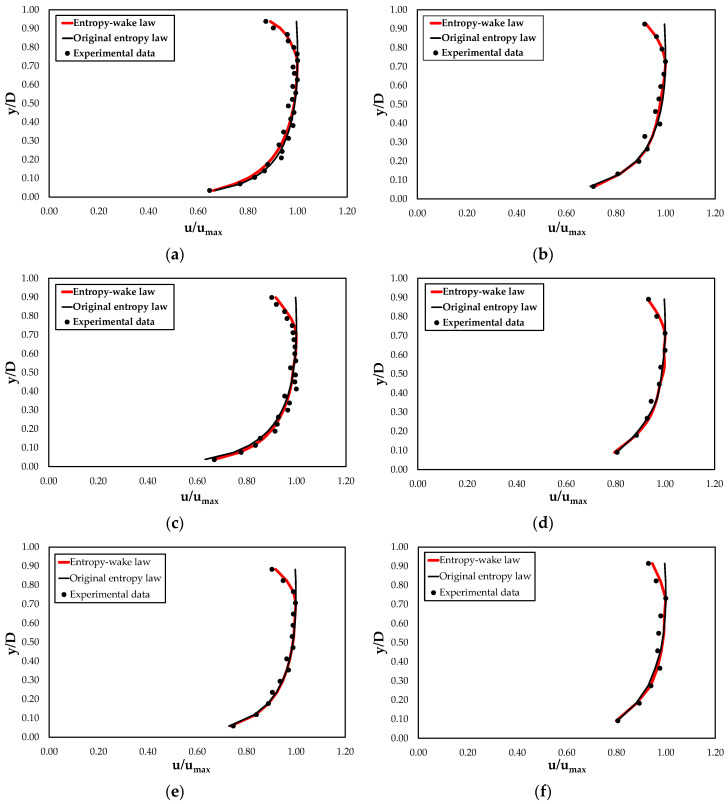
Comparison of velocity profiles computed through entropy wake law and original entropy law with the experimental data for different values of the aspect ratio (*A_r_*) and bottom slope (*i*): (**a**) *A_r_* = 3 and *i* = 0.00%; (**b**) *A_r_* = 7 and *i* = 0.00%; (**c**) *A_r_* = 4 and *i* = 0.50%; (**d**) *A_r_* = 9 and *i* = 0.50%; (**e**) *A_r_* = 4 and *i* = 1.00%; (**f**) *A_r_* = 9 and *i* = 1.00%.

**Figure 7:**
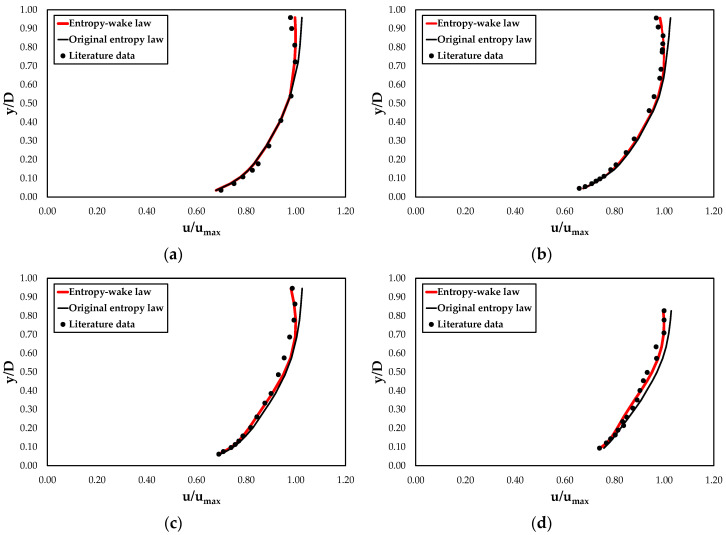
Comparison of the velocity profiles computed through the entropy wake law and through the original entropy law with the literature data: (**a**) Coleman [16] RUN21; (**b**) Lyn [78] C1; (**c**) Lyn [76] C3; (**d**) Muste and Patel [79] CW02.

Figure 6 and Figure 7 report the predicted velocity profiles through the entropy wake and classical entropy laws with the experimental data collected in this research and in the literature. As noted in the figures, although both models agree very well with the velocities near the channel bed, the original entropy model is not able to describe the flow field in the outer region close to the free surface. In particular, the latter tends to overestimate the measured data and this deviation increases with a decreasing aspect ratio. This condition could be especially due to a greater effect of the secondary currents and the sidewalls on the streamwise velocity profile.

**Figure 8:**
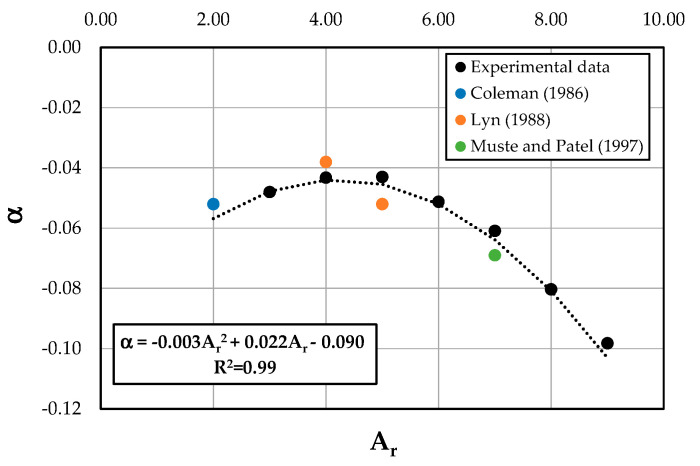
Relationship between *α* and *A_r_* along the centerline profile of the rectangular channel.

The proposed entropy wake law, instead, thanks to the presence of additional terms, reproduces perfectly the dip phenomenon and gives a good description of the velocity distribution over the entire water column, including the inner and outer regions. Only a light underestimation of the observed velocities was noted for lower values of the *A_r_*. This suggests investigating the dependence of the coefficient *α* on the aspect ratio. Therefore, Figure 8 reports the relationship between *α* and *A_r_* based on the experimental data. It is possible to see from the figure that the data interpolation shows how *α* could be a quadratic function of the aspect ratio equal to

(22) $$\alpha =-0.003A_{r}^{2}+0.022A_{r}-0.090.$$

Figure 8 displays also how the coefficient *α* tends to decrease for the condition of a wider channel when the dip phenomenon disappears, while it increases when the influence of the secondary flows is strong. However, the effect of the secondary currents on the value of the coefficient *α* cannot be described only by the aspect ratio. Therefore, a more detailed investigation of flow conditions and channel characteristics is needed to validate the obtained empirical relationship.

## 5. Conclusions

A new wake law for the estimation of streamwise velocity profiles in steady open channel flows was derived from Shannon’s informational theory together with the principle of maximum entropy. The proposed law includes three additional terms: the first similar to Coles’ function; the second linearly proportional to the logarithmic distance from the free surface; and the third depending on the cubic correction near the maximum velocity. Although the entropy wake law presents a more complex equation compared with that of the classical entropy profile, it depends on a single coefficient, *α*, which seems to keep constant in all three terms.

A set of laboratory measurements was carried out in a rectangular straight flume, with a smooth bed and sidewalls, for different values of water discharge, aspect ratio, and bottom slope, in order to validate the developed formula. A detailed error analysis demonstrated the very good performance of the modified entropy law in predicting the flow field over the entire water column, including the inner and outer regions. In addition, a further validation of the proposed model was carried out using the literature data and it confirmed the high accuracy of the new law.

The comparison with the streamwise velocity profiles calculated through the classical entropy model showed how the velocity distributions obtained from the proposed formula are able to reproduce the dip phenomenon and the velocity negative gradient near the free surface, as well as to match the experimental and literature data perfectly. Actually, a light underestimation of the entropy wake law was observed for low values of the aspect ratio, which led to examine the dependence of the coefficient *α* on the *A_r_*. Such a study, although still in a preliminary phase, demonstrated that the presence of secondary currents and the effects of sidewalls affect the parameter *α*. However, a deeper analysis of this parameter is needed in the future. The high precision of the new model in describing correctly the streamwise velocity distributions of a smooth and rectangular cross-section suggests that it could also be used to investigate flows in open channels with different shapes and boundary roughness conditions.

## Figures and Tables

**Table 1:** Mean values and variances of sampled signals with varying time.

		t = 10 s	t = 20 s	t = 30 s	t = 40 s	t = 50 s	t = 60 s
**Low velocity**	μ (m/s)	0.24	0.24	0.24	0.24	0.24	0.24
	σ^2^ (m^2^/s^2^)	0.00014	0.00013	0.00013	0.00012	0.00011	0.00010
**High velocity**	μ (m/s)	1.20	1.21	1.21	1.21	1.21	1.21
	σ^2^ (m^2^/s^2^)	0.00024	0.00030	0.00034	0.00034	0.00031	0.00029

**Table 2:** Ranges of the flow characteristics of the laboratory tests.

*i* (%)	*Q* (m^3^/s)	*D* (m)	*A_r_*	*u_mean_* (m/s) ^1^	*u_max_* (m/s) ^2^
0	0.010–0.095	0.053–0.204	3.0–9.0	0.36–1.20	0.489–1.459
0.25	0.018–0.085	0.057–0.171	3.0–9.0	0.63–1.27	0.824–1.582
0.50	0.023–0.084	0.056–0.140	4.0–9.0	0.77–1.32	0.974–1.576
0.75	0.026–0.085	0.058–0.134	4.0–9.0	0.86–1.29	1.092–1.551
1.00	0.026–0.077	0.055–0.116	4.0–9.0	0.50–0.91	0.621–1.308

^1^*u_mean_* is the mean velocity of the cross-section. ^2^ *u_max_* is the maximum velocity of the cross-section.

**Table 3:** Performance ratings for suggested model evaluation statistics.

Statistics	Performance Rating			
	Very Good	Good	Satisfactory	Unsatisfactory
**RSR**	0.00 ≤ RSR ≤ 0.50	0.50 < RSR ≤ 0.60	0.60 < RSR ≤ 0.70	RSR > 0.70
**PBIAS**	PBIAS < ±10	±10 ≤ PBIAS < ±15	±15 ≤ PBIAS < ±25	PBIAS ≥ ±25
**NSE**	0.75 < NSE ≤ 1.00	0.65 < NSE ≤ 0.75	0.50 < NSE ≤ 0.65	NSE ≤ 0.50

**Table 4:** Comparison between the statistical indices of velocities computed through the entropy wake law and through the classical entropy model for the experimental data acquired in this research.

Statistical Indices	Entropy Wake Law	Classical Entropy Law
**RMSE**	0.04	0.14
**MAE**	2.52	13.07
**RSR**	0.15	0.66
**PBIAS**	0.61	10.41
**NSE**	0.98	0.56

**Table 5:** Geometric and kinematic characteristics of the literature data in the conditions of clear water.

Data Set	Coleman (1986)			Lyn (1988)				Muste and Patel (1997)		
	RUN1	RUN21	RUN32	C1	C2	C3	C4	CW01	CW02	CW03
*i* (%)	0.200	0.2	0.2	0.206	0.270	0.296	0.401	0.0741	0.0768	0.0813
*Q* (m^3^/s)	0.064	0.064	0.064	0.011	0.013	0.011	0.013	0.0738	0.0735	0.0733
*D* (m)	0.172	0.169	0.173	0.0654	0.0653	0.0575	0.0569	0.130	0.128	0.127
*A_r_*	2.07	2.11	2.06	4.08	4.09	4.64	4.69	7.00	7.11	7.16
*u_max_* (m/s)	1.06	1.05	1.03	0.753	0.875	0.857	1.019	0.700	0.708	0.715

**Table 6:** Comparison between the statistical indices of the velocities computed through the entropy wake law and those computed through the classical entropy model for the literature data.

	Statistical Indices	Entropy Wake Law	Classical Entropy Law
**Coleman (1986)**	**RMSE**	0.03	0.11
	**MAE**	1.05	4.56
	**RSR**	0.18	0.72
	**PBIAS**	1.02	10.96
	**NSE**	0.96	0.54
**Lyn (1988)**	**RMSE**	0.03	0.16
	**MAE**	1.15	6.12
	**RSR**	0.21	0.77
	b	1.67	11.37
	**NSE**	0.95	0.48
**Muste and Patel (1997)**	**RMSE**	0.09	0.22
	**MAE**	1.45	7.01
	**RSR**	0.30	0.97
	**PBIAS**	1.94	12.12
	**NSE**	0.83	0.46
